# Molecular Dynamics Simulation of Hydrogen Barrier Performance of Modified Polyamide 6 Lining of IV Hydrogen Storage Tank with Graphene

**DOI:** 10.3390/polym16152185

**Published:** 2024-07-31

**Authors:** Jin Li, Xiaokou Zhao, Jianguo Liang, Chunjiang Zhao, Ning Feng, Guanyu Guo, Zhengze Zhou

**Affiliations:** 1 College of Mechanical Engineering, Taiyuan University of Science and Technology, Taiyuan 033024, Chinas202112210646@stu.tyust.edu.cn (G.G.);; 2 Department of Resource and Mechanical Engineering, Lyuliang University, Lvliang 033001, China; 3 College of Mechanical and Vehicle Engineering, Taiyuan University of Technology, Taiyuan 030024, China; 4 College of Intelligent Manufacturing Industry, Shanxi University of Electronic Science and Technology, Linfen 041000, China

**Keywords:** polyamide 6, graphene, hydrogen diffusion, molecular dynamics, free volume

## Abstract

The polymer liner of the hydrogen storage cylinder was studied to investigate better hydrogen storage capacity in Type-IV cylinders. Molecular dynamics methods were used to simulate the adsorption and diffusion processes of hydrogen in a graphene-filled polyamide 6 (PA6) system. The solubility and diffusion characteristics of hydrogen in PA6 systems filled with different filler ratios (3 wt%, 4 wt%, 5 wt%, 6 wt%, and 7 wt%) were studied under working pressures (0.1 MPa, 35 MPa, 52 MPa, and 70 MPa). The effects of filler ratio, temperature, and pressure on hydrogen diffusion were analyzed. The results show that at atmospheric pressure when the graphene content reaches 5 wt%, its permeability coefficient is as low as 2.44 × 10^−13^ cm^3^·cm/(cm^2^·s·Pa), which is a 54.6% reduction compared to PA6. At 358 K and 70 MPa, the diffusion coefficient of the 5 wt% graphene/PA6 composite system is 138% higher than that at 298 K and 70 MPa. With increasing pressure, the diffusion coefficients of all materials generally decrease linearly. Among them, pure PA6 has the largest diffusion coefficient, while the 4 wt% graphene/PA6 composite system has the smallest diffusion coefficient. Additionally, the impact of FFV (free volume fraction) on the barrier properties of the material was studied, and the movement trajectory of H_2_ in the composite system was analyzed.

## 1. Introduction

Hydrogen is a crucial component of global decarbonization strategies due to its carbon-free, efficient, and renewable nature. It will play a central role in the ongoing development and successful transition of traditional energy systems, effectively mitigating the negative impacts of carbon dioxide emissions, such as global warming [1,2]. In the transportation sector, hydrogen fuel cell vehicles (HFCVs) have been extensively researched. Many companies, including Toyota, Honda, and Hyundai, are dedicated to developing high-performance hydrogen fuel cell vehicles [3].

Hydrogen, as fuel for hydrogen fuel cell vehicles, is typically compressed and stored in pressure vessels, which is currently the mainstream technology for onboard hydrogen storage. Among these, Type III and Type IV pressure vessels are the most widely used [4,5]. Compared to Type III hydrogen storage cylinders with metal liners, Type IV hydrogen storage cylinders use polymer liners [6], including high-density polyethylene (HDPE), polyamide (PA), polyethylene terephthalate (PET), and various polyether materials. These Type IV cylinders offer numerous advantages, such as high hydrogen storage density, lightweight, corrosion resistance, and fatigue resistance [7,8].

Polyamide (PA6) has gradually become a potential choice for Type IV hydrogen storage vessel liners due to its strong molecular polarity and hydrogen bonding interactions [9,10]. However, Type IV hydrogen storage vessels face challenges where the polymer liner directly contacts hydrogen while being bonded to an external fiber-wound layer. In high-pressure hydrogen environments, hydrogen permeates into the material, diffuses to the plastic-composite interface, and creates internal pressure. Upon depressurization, hydrogen escapes from the material to the exterior, creating a pressure differential at the interface, which leads to liner deformation and bulging [11,12]. Understanding the hydrogen transport properties of polymers under harsh operating conditions (233–358 K, 0–87 MPa) is crucial [13,14]. Some researchers have studied polymer permeability to hydrogen, focusing on external conditions (including temperature [15,16] and pressure [17,18]), gas-material interactions [19,20], material properties (including crystallinity [21,22,23], polymer molecular weight [20,24], filler structure [23,25,26], filler content [23,27,28]), and others. However, further research is needed to understand the hydrogen permeation characteristics of PA6 fully.

Using the Einstein relation [29], the molecular dynamics (MD) software Materials Studio (2019) is employed to simulate the structure-property relationships and study the diffusion coefficients of small gas molecules in polymer material models. This method effectively predicts the hydrogen permeability of polymer liners in hydrogen storage cylinders [20,30]. Wu [31] investigated the adsorption and diffusion processes of hydrogen in PA6 systems filled with modified montmorillonite (OMMT) under different filler contents (3–7%), temperatures (288–328 K), and pressures (0–60 MPa). The results indicated that at a filler content of 5%, the material’s permeability coefficient was less than 2 × 10^−13^ cm^3^·cm/(cm^2^·s·Pa). Additionally, as the pressure increased, the permeability coefficient of the modified material first decreased and then increased. Su [14] comprehensively explored the dissolution and diffusion behavior of H_2_ in PA6 under service conditions (233–358 K, 0–87 MPa), finding that the diffusion coefficient and permeability coefficient were positively correlated with temperature, while the solubility coefficient was also positively correlated with temperature. MURARU [28] evaluated the gas permeability of PSF-cnt-g membranes and compared them with three other membranes (PSF, PSF-cnt, and PSF-g), discovering that the addition of carbon nanotubes and graphene to polysulfone membranes significantly increased the diffusion coefficients of gases such as CH_4_, CO_2_, H_2_, N_2_, and O_2_. Yi [23] studied the diffusion of gases, including hydrogen and its isotopes, at room temperature and pressure, noting that the diffusion decreased with increasing polystyrene molecular weight. Zhang [32] analyzed the diffusion characteristics of hydrogen in HDPE under temperature (room temperature to 80 °C) and pressure (2.5–10 MPa) conditions. The results showed that when the temperature increased from 30 °C to 80 °C, hydrogen’s solubility, diffusion coefficient, and permeability coefficient in HDPE increased by 18.7%, 92.9%, and 129.0%, respectively. Fang [12] simulated the diffusion and adsorption processes of hydrogen molecules in polyethylene (PE) and PA6 at temperatures ranging from 263 K to 353 K. The results indicated that under the same conditions, the solubility, diffusion, and permeability coefficients of hydrogen in PE were higher than those in PA6, suggesting that PA6 has better hydrogen barrier properties than PE. Hu [33] studied the diffusion characteristics and local structure of a mixed system composed of CH_4_, CO_2_, SO_2_, and H_2_O, finding that higher temperatures and lower pressures favored gas diffusion.

Previous studies have shown that the diffusion characteristics of polymers are related to additives, different temperatures, and pressures. Although the aforementioned simulations focus on gas diffusion in various polymers under different temperatures and pressures, the impact of graphene-modified PA6 on H₂ diffusion at extreme temperatures (233 K–358 K) and operating pressures of gas cylinders (0.1–70 MPa) has not been systematically investigated.

Materials Studio (2019) was used in this study to construct a molecular model of a composite material with PA6 as the polymer liner and graphene as the nanofiller. Analyzing the permeation behavior of H_2_ in modified PA6 materials from a microscopic perspective to elucidate the characteristics and mechanisms of hydrogen permeation seems to be a very interesting endeavor. This approach can provide a method for evaluating the performance of liner materials for Type IV hydrogen storage cylinders. Our research aims to offer more evidence for the selection of liner materials for hydrogen storage cylinders.

## 2. Model and Simulation Methods

### 2.1. Establishment of Modified Liner Models

The MD simulation study utilized amorphous unit cells to construct polymer liners. Single-chain structures were built using the Builder module, and monomer structures were constructed using the Sketch toolbar, establishing 60 repeated units to form random single-chain molecules of PA6. The AC module was employed to generate models of five different systems, each containing 20 H_2_ molecules and identical PA6 chains of 8 repeated units [28,31,34]. Hydrogen positions were randomly distributed to investigate their diffusion within the liner models.

**Figure 1:**
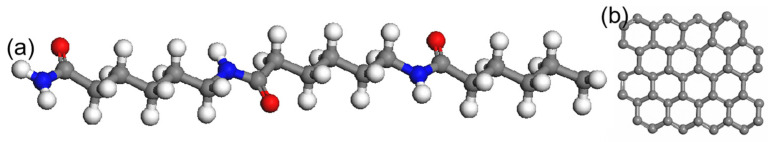
(**a**) PA6 repeating unit (gray atoms—carbon, white atoms—hydrogen, red atoms—oxygen, blue atoms—nitrogen). (**b**) Graphene sheet.

**Figure 2:**
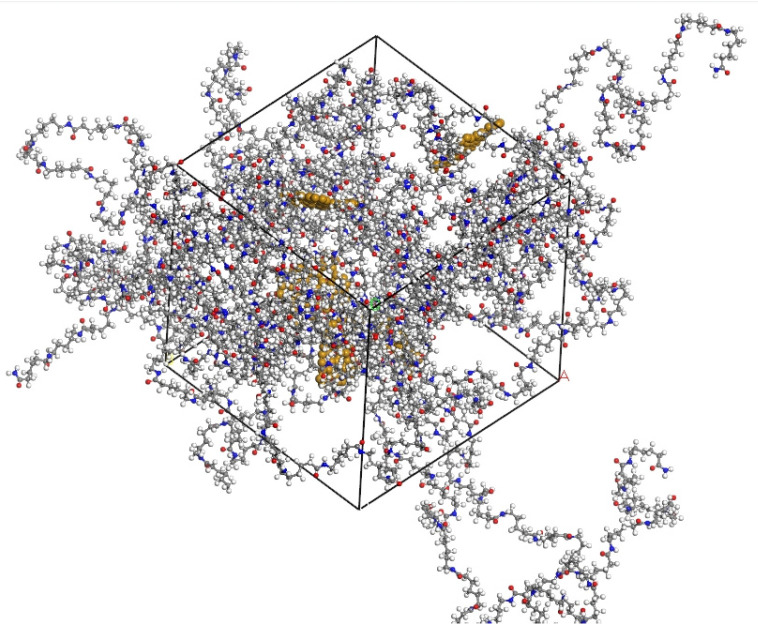
Model of graphene-modified PA6 (yellow atom-graphene sheet).

In the composite model systems, graphene, initially composed of single sheets containing 48 carbon atoms, was introduced. Subsequently, the graphene mass fraction was varied by gradually increasing the number of graphene sheets to achieve mass fractions of 3 wt%, 4 wt%, 5 wt%, 6 wt%, and 7 wt%. The unit cell dimensions for different mass fractions were approximately 44.06 Å, 44.11 Å, 44.06 Å, 44.32 Å, and 44.40 Å, with identical lattice dimensions in the a, b, and c directions for all unit cells. Figure 1 illustrates the structures of PA6 chains and graphene. Figure 2 depicts the construction of mixed models of PA6 chains and graphene using the random copolymer option.

In the composite model systems, graphene, initially composed of single sheets containing 48 carbon atoms, was introduced. Subsequently, the graphene mass fraction was varied by gradually increasing the number of graphene sheets to achieve mass fractions of 3 wt%, 4 wt%, 5 wt%, 6 wt%, and 7 wt%. The unit cell dimensions for different mass fractions were approximately 44.06 Å, 44.11 Å, 44.06 Å, 44.32 Å, and 44.40 Å, with identical lattice dimensions in the a, b, and c directions for all unit cells. Figure 1 illustrates the structures of PA6 chains and graphene. Figure 2 depicts the construction of mixed models of PA6 chains and graphene using the random copolymer option.

### 2.2. Simulation Method

The initial models underwent geometric optimization using the Smart method with energy minimization applied to all monomers and models to eliminate local non-equilibrium states. The maximum iteration step was set to 5 × 10^4^ to achieve stability across all models. Subsequently, a cyclic annealing process was applied to the polymer material models to obtain more realistic polymer structures. According to the simulated annealing method, starting from 300 K and ramping up to a midpoint temperature of 600 K, a total of 30 annealing cycles were conducted. This thermal annealing reduced internal stresses in the modified liner models and nearly eliminated any structurally unreasonable configurations generated during optimization.

The optimized models were subjected to molecular dynamics (MD) simulations using the COMPASS II force field [35]. The simulations were conducted in the NVT [12,14,20] ensemble at an initial temperature of 298 K for a relaxation period of 1 ns. The MD simulations aimed to stabilize the energy and density of the entire system. During MD simulations, the Andersen temperature control method and Berendsen pressure control were applied to maintain the models’ constant temperature [36,37,38]. The Group-Based [39] method was utilized to calculate non-bonded interactions and Coulomb forces. Integration of the equations of motion was performed with a time step of 1 fs for all simulations [38,39,40,41].

**Figure 3:**
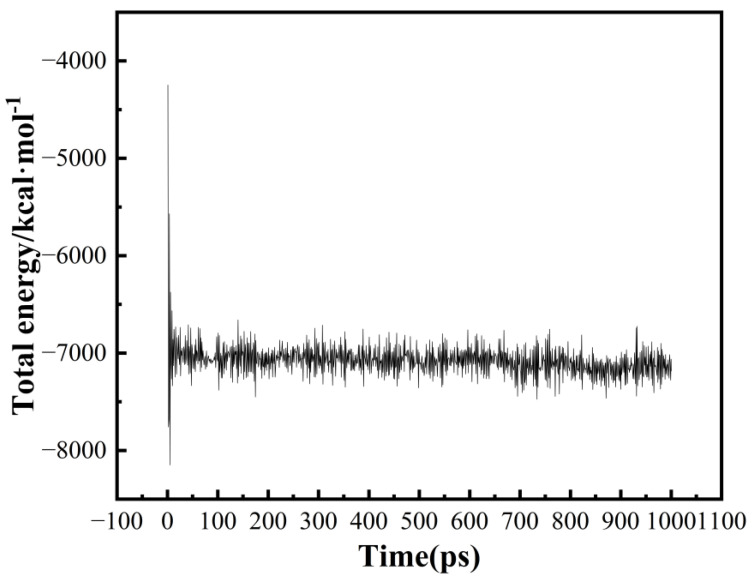
Total energy changes in the PA6 model during dynamic treatment.

Figure 3 illustrates the change in total energy during the dynamic processing. Over time, the total energy of the system stabilizes, oscillating within a narrow range around a fixed value. This indicates that the models have achieved full relaxation and obtained stable structures.

### 2.3. Model Reliability Verification

**Figure 4:**
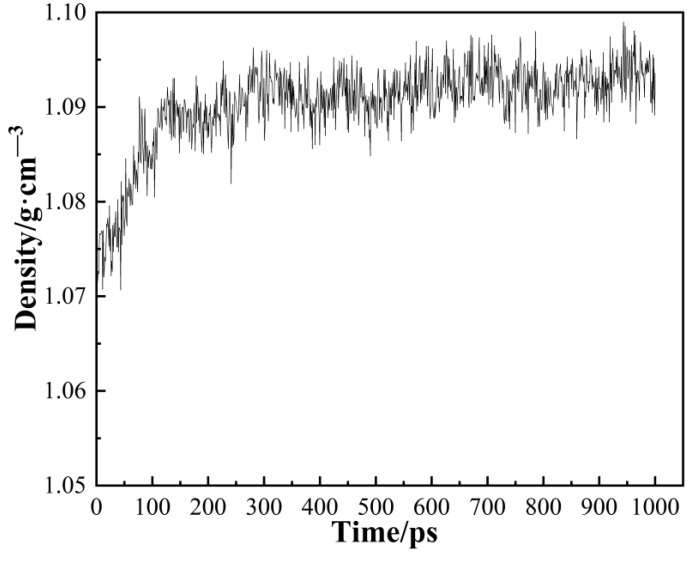
The density of PA6 during the relaxation process (Conditions: 298 K and 0.1 MPa).

Taking PA6 as an example, it exhibits a fully amorphous structure. Figure 4 depicts the density variations observed throughout the relaxation process. It can be observed that the final density of PA6 is 1.095 g/cm³. Comparing this with the actual density of 1.13 g/cm³ for PA6 in an amorphous state, the relative error is calculated to be 3.1%, indicating the validity of the models proposed in this study.

The solubility coefficients of H_2_ in the six systems were obtained using adsorption isotherms within the pressure range of 0.01 kPa to 10,000 kPa. According to the Chinese National Standard (GB/T 42612-2023) [42], Type IV hydrogen storage cylinders operate at a maximum working pressure of 70 MPa, with a working temperature range not lower than −40 °C and not higher than 85 °C. Exceeding this temperature range can cause irreversible damage to the carbon fibers in the gas cylinder. Therefore, diffusion analysis was conducted at three temperatures: 233 K, 298 K, and 358 K. Therefore, to determine the diffusion coefficients under different conditions, pressures of half the maximum working pressure of Type IV hydrogen storage cylinders (35 MPa) and an intermediate value of 52.5 MPa (between 35 MPa and 70 MPa) were selected. The model was subjected to four different pressures (0.1 MPa, 35 MPa, 52.5 MPa, and 70 MPa). One ns molecular dynamics simulations were performed under the NPT ensemble for six different systems. The mean square displacement (MSD) curves of hydrogen molecules were calculated, and the diffusion coefficients of H_2_ were obtained.

## 3. Theoretical Basis of Permeability

### 3.1. Adsorption Concentration

The determination of the solubility coefficient (S) of gas molecules in polymers can be achieved through the application of adsorption isotherms. Under constant temperature conditions, adsorption isotherms are derived based on the concentration corresponding to different solubilities. Henry’s law applies to systems with the smallest solubility and can be used to explain the dissolution process of small gas molecules such as hydrogen in polymers [41,43]. This relationship is expressed in Equation (1). It is noteworthy that when the fugacity approaches zero, the solubility coefficient can be determined as the limiting slope of the adsorption isotherm, as shown in Equation (2).

(1) $$C=K_{D}P+C_{H}\frac{bP}{1+bP}$$

(2) $$S=\underset{P\to 0}{\lim}\frac{C}{P}=K_{D}+C_{H}P$$

where *K_D_* is the Henry constant, *C_H_* is the Langmuir capacity parameter, *b* is the Langmuir parameter, *P* is the pressure, and *C* is the adsorption of gas molecules.

### 3.2. Diffusion Coefficient

The diffusion coefficient (D) describes the Brownian motion of particles under concentration gradient conditions. It represents the dynamic characteristics of interaction between permeable gas molecules and polymers. The diffusion coefficient D can be determined by analyzing the MSD of molecular motion. In MD simulations, the correlation between MSD and molecular motion time is established by tracking the motion trajectory of permeating molecule centers. Subsequently, the diffusion coefficient is calculated using the Einstein formula [24], as shown in Equation (3):

(3) $$D=\frac{1}{6N}\underset{t\to \infty }{\lim}\frac{d}{dt}\langle \sum_{i}^{N}{\left[r_{i}\left(t\right)-r_{i}\left(0\right)\right]}^{2}\rangle =\frac{a}{6}$$

where *D* is the diffusion coefficient, $r_{i}\left(t\right)$ and $r_{i}\left(0\right)$ are the position vectors of molecule *i* at times *t* and 0, respectively. *N* represents the total number of gas particles, and $\langle {\left[r_{i}\left(t\right)-r_{i}\left(0\right)\right]}^{2}\rangle$ denotes the ensemble average of the MSD of gas molecules. Gas molecules collide within a small pocket of free volume, jumping from one confined region to another; the repeated jumps of molecules constitute diffusion, which is characterized by the MSD, and a represents the gradient of the MSD curve obtained from molecular simulations.

### 3.3. Permeability Coefficient

The “dissolution-diffusion” theory can describe the permeation process of gas molecules in polymers. Gas molecules dissolve from the surface into the polymer and diffuse from one side of the polymer to the other. Finally, gas molecules desorb and escape from the polymer [44]. Therefore, the permeation process of H_2_ in polymers can be divided into dissolution and diffusion processes. The permeability coefficient [45] is the product of the solubility and diffusion coefficients, as shown in Equation (4).

P = S × D(4)

where P is permeability coefficient, cm^3^·cm/(cm^2^·s·Pa), S is the solubility coefficient, cm^3^·cm^−3^·Pa^−1^, and D is the diffusion coefficient, cm^2^/s.

### 3.4. Free Volume

To obtain the free fraction volume (FFV) of the polymer [12,31,46], an analysis of the five models was conducted using the Atom V volume and Surface tools. The Connolly surface of the system was calculated based on the known van der Waals radius of hydrogen atoms, with a Connolly radius of 1.4 Å, to determine the system’s FFV.

## 4. Results and Discussion

### 4.1. Effect of Filler Ratio on Permeation Coefficient

**Figure 5:**
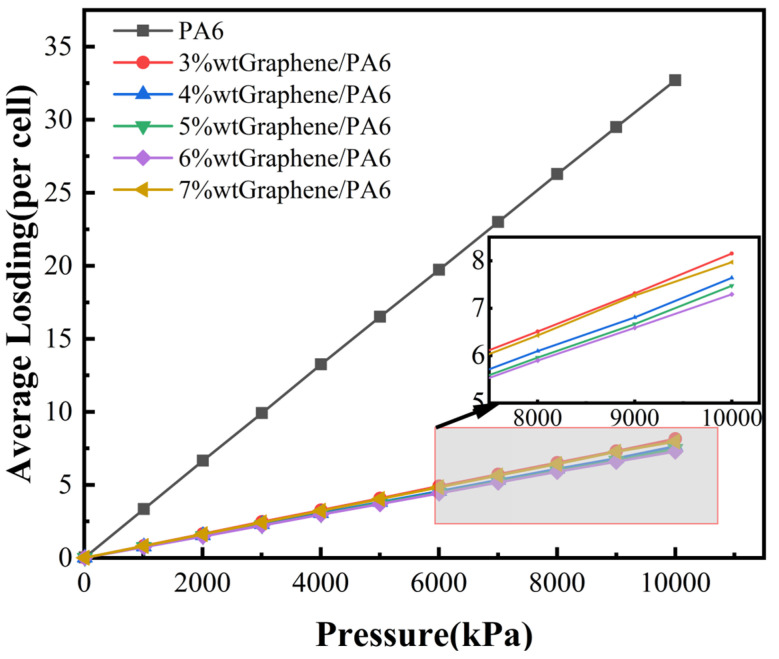
Isothermal Adsorption Curves of H_2_ in Different Systems at 298 K.

**Figure 6:**
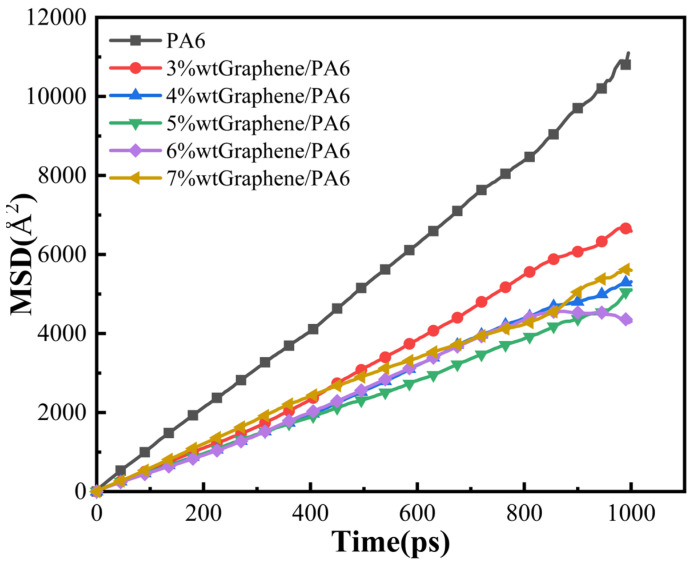
MSD Curves of H_2_ in Different Systems (Conditions: 298 K and 0.1 MPa).

Figure 5 and Figure 6 illustrate the isothermal adsorption curves and MSD curves of H_2_ in various graphene/PA6 systems under conditions of 298 K and 0.1 MPa. The simulated data were fitted, and solubility coefficient S and diffusion coefficient D were calculated using Equations (2) and (3), respectively. Permeability coefficient P was then derived using Equation (4), and the calculated results are presented in Table 1.

Based on Figure 5, it is evident that the adsorption concentration of H_2_ in the polymer shows an approximately linear relationship with pressure. Additionally, PA6 exhibits the highest adsorption concentration. Combined with Table 2, it is observed that the solubility coefficient varies with the addition of graphene in different graphene/PA6 composite systems. When the graphene content reaches 6 wt%, the adsorption of H_2_ decreases to its minimum, with a solubility coefficient of 3.01 × 10^−7^ cm^3^·cm^−3^·Pa^−1^, representing a reduction of 77.2% compared to pure PA6. The solubility coefficient reflects the thermodynamic interactions between gas molecules and polymer chains. According to the free volume theory, permeating gas molecules generally occupy less dense regions within the polymer, known as free volume, through physical adsorption. With the addition of graphene filler, interactions between the filler and PA6 chains restrict the mobility of polymer chains, thereby reducing the formation of free volume within the system. Consequently, the available area for gas adsorption within the material decreases, impacting its permeability to leaking gases.

At a content of 3 wt%, the solubility coefficient reaches its maximum at 3.33 × 10^−7^ cm^3^·cm^−3^·Pa^−1^, which is not significantly different from the solubility coefficient of the 6 wt% graphene/PA6 composite system. This suggests that when the graphene mass fraction exceeds 3 wt%, the solubility coefficient is not the primary factor influencing the permeation performance of H_2_ in the composite system. This finding is consistent with the results reported by Zheng [47].

Figure 6 shows that the MSD curve exhibits a linear relationship with time. Before Einstein diffusion, anomalous diffusion may occur due to structural reasons within the system. It is essential to characterize the system diffusion properties to verify if the system has undergone normal diffusion.

**Figure 7:**
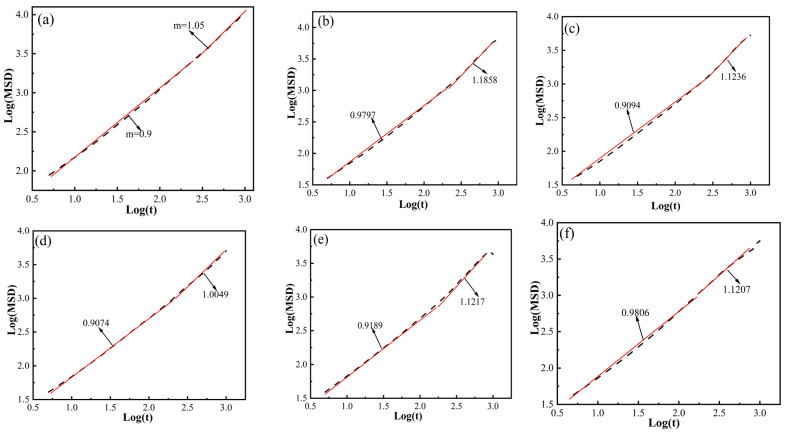
Logarithmic Plot of H_2_ MSD vs. Simulation Time at 298 K and 0.1 MPa: (**a**) PA6, (**b**) 3 wt%, (**c**) 4 wt%, (**d**) 5 wt%, (**e**) 6 wt%, and (**f**) 7 wt%.

The verification can be conducted by examining the slope of the logarithmic plot of MSD against time [48,49]. Figure 7 depicts the logarithmic plot log(MSD) vs. log(t) for determining diffusion coefficients using the Einstein relationship. Here, the log(MSD) vs. log(t) curve primarily assesses whether H_2_ molecules in the graphene/PA6 composite systems transition from anomalous diffusion (K < 1) to normal diffusion (K > 1). It is observed that H_2_ undergoes this transition in various graphene/PA6 composite systems with different filler ratios, allowing for the calculation of diffusion coefficients using the Einstein relationship.

The diffusion coefficient reflects the dynamic interactions between gas molecules and polymer chains. Figure 6 and Table 1 demonstrate that the introduction of graphene reduces the diffusion coefficients of H_2_ in all systems. As the proportion of graphene increases, the diffusion coefficients initially decrease and then increase, which affects the permeability of the materials. Specifically, when graphene reaches 5 wt%, the composite system exhibits the lowest diffusion coefficient, leading to the minimum permeability. At this composition, the permeability of the 5 wt% graphene/PA6 composite for H_2_ is as low as 2.44 × 10^−13^ cm^3^·cm/(cm^2^·s·Pa), indicating optimal barrier properties under these conditions. Compared to pure PA6, the permeability of the 5 wt% graphene/PA6 composite is reduced by 54.6%. The reasons behind these results may be attributed to several factors. Firstly, the introduction of a small amount of filler disrupts the continuity of “pores” within the system to some extent, forming barriers (or “walls”). When H_2_ molecules move within the polymer matrix, they encounter these graphene filler barriers, increasing the energy required for diffusion and complicating the diffusion pathways. This restriction limits the diffusion of H_2_ molecules. Secondly, the presence of graphene reduces the free volume within the system. However, when the filler content is too high (e.g., 6 wt%), the randomly arranged graphene interacts with the polymer to form new “pores,” increasing the internal free volume and consequently increasing the diffusion coefficient of H_2_ molecules.

### 4.2. Impact of Temperature Diffusion Coefficients

**Figure 8:**
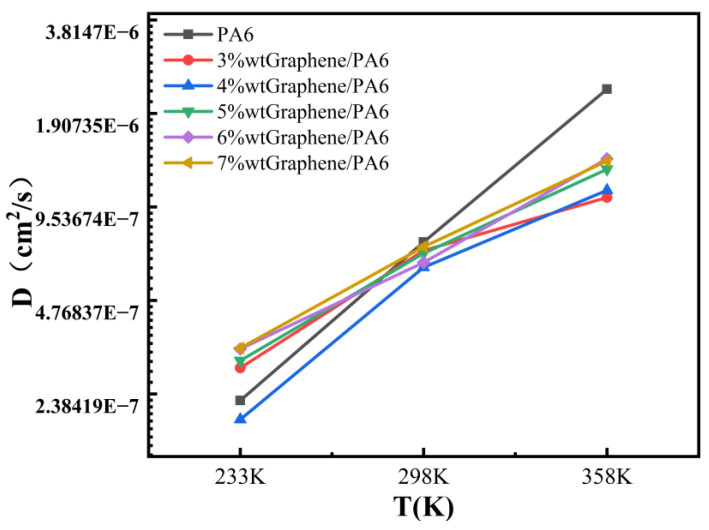
Diffusion coefficients of six systems at different temperatures.

Figure 8 and Table 2 present the diffusion coefficients of six systems at three different temperatures (233 K, 298 K, and 358 K). The results indicate that at 358 K and 70 MPa pressure, PA6 exhibits the highest diffusion coefficient of 2.28 × 10^−6^ cm^2^/s. As shown in Figure 8, the diffusion coefficient of PA6 increases correspondingly with temperature. This phenomenon can be attributed to the energy required for hydrogen molecules to overcome inter-molecular interactions within the polymer matrix during their diffusion. At higher temperatures, the kinetic energy of polymer chains increases, facilitating greater mobility of hydrogen molecules within the polymer structure [10,50]. Therefore, the diffusion coefficient increases with temperature.

It is important to emphasize that Dong [8] experimentally measured the diffusion coefficient of PA6 at 358 K and 87.5 MPa to be 2.36 × 10^−6^ cm^2^/s. Therefore, we specifically analyzed the diffusion coefficient of PA6 under these conditions, resulting in 2.11 × 10^−6^ cm^2^/s. By comparing these two values, we found the error within 10.6%. In the context of gas diffusion coefficients at the small magnitude of 10^−7^, errors within 20% are generally considered reasonable. Considering the crystallinity of PA6, these findings demonstrate that molecular dynamics simulations can effectively approximate the hydrogen barrier properties of the material. This also validates the reliability of the research methods and molecular dynamics simulation techniques used in this study.

Figure 8 and Table 2 show that the diffusion coefficients of all systems increase with temperature at 358 K compared to 233 K and 298 K. Specifically, the diffusion coefficient of 3 wt% graphene/PA6 at 358 K is 1.02 × 10^−6^ cm^2^/s, which is 48% higher than at 298 K. Similarly, the diffusion coefficients of 4 wt% to 7 wt% graphene/PA6 increase by approximately 77%, 138%, 115%, and 89%, respectively, at 358 K compared to 298 K. This illustrates the significant influence of temperature on the diffusion coefficients of the materials, a phenomenon supported by previous studies [41,42].

### 4.3. Effect of Pressure on Diffusion Coefficients

Figure 5 demonstrates that at constant temperature, the adsorption of H_2_ in the materials is approximately proportional to pressure. The solubility of H_2_ in the materials shows almost no change with pressure; thus, pressure variations have minimal impact on the materials’ solubility coefficient. Gas solubility is considered constant at different pressures because pressure does not affect the thermodynamic properties of gas molecules and polymers. Dong [8] analyzed the influence of different test temperatures and pressures on the solubility coefficient of PA6, noting a complex interaction between temperature and gas activity capacity affecting H_2_ solubility in PA6. This results in limited effects of test temperature and pressure on the solubility coefficient. Fujiwara [50] conducted permeability coefficient experiments on HDPE under pressures ranging from 10 to 90 MPa, indicating that solubility coefficients do not significantly depend on pressure; changes in permeability coefficients are primarily diffusion-controlled.

**Figure 9:**
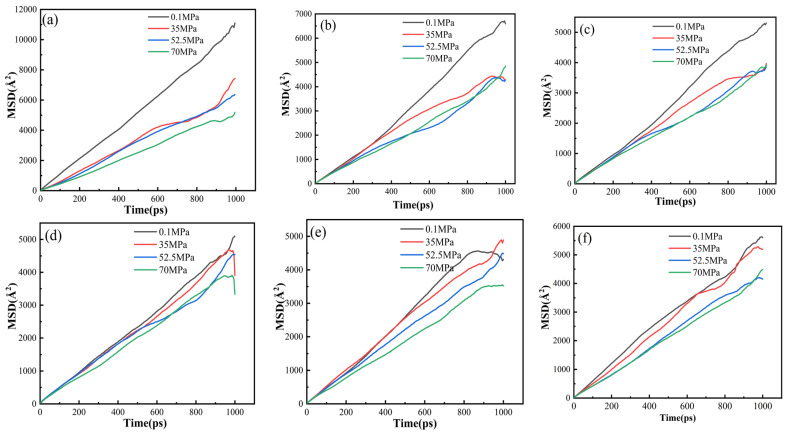
MSD Curves of Different Graphene Content Systems at 298 K: (**a**) PA6, (**b**) 3 wt%, (**c**) 4 wt%, (**d**) 5 wt%, (**e**) 6 wt%, and (**f**) 7 wt%.

**Figure 10:**
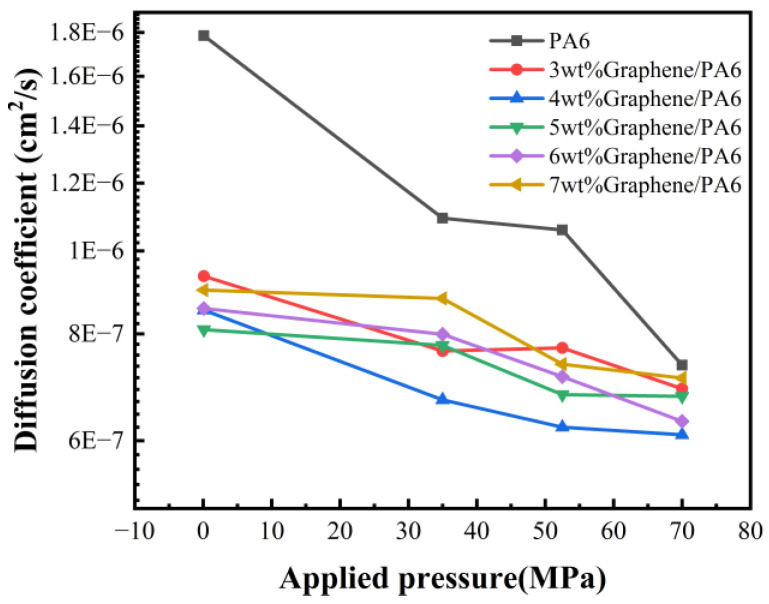
Diffusion Coefficients of 6 Systems at 298 K under Different Pressures.

Figure 9 illustrates the MSD curves of six material systems at 298 K under different pressures. It can be observed that as the pressure increases, the slope of the curves gradually decreases, indicating a significant influence of pressure on the diffusion characteristics of the materials. Figure 10 shows the calculated diffusion coefficients under different pressures, demonstrating a substantial impact of pressure on the diffusion of H_2_ within the systems. As pressure increases, the diffusion coefficients of all materials generally decrease linearly. Specifically, pure PA6 exhibits the highest diffusion coefficient, while the 4 wt% graphene/PA6 composite system shows the lowest diffusion coefficient.

Table 3 presents the permeability coefficients of different systems at 298 K under various pressures. It can be observed that within the same system, permeability decreases with increasing pressure. At 0.1 MPa pressure, the permeability of 5 wt% graphene/PA6 is the lowest, at 2.44 × 10^−13^ cm^3^·cm/(cm^2^·s·Pa). At pressures above 35 MPa, the permeability of the 4 wt% graphene/PA6 is the lowest, with values of 2.15 × 10^−13^ cm^3^·cm/(cm^2^·s·Pa), 2.00 × 10^−13^ cm^3^·cm/(cm^2^·s·Pa), and 1.96 × 10^−13^ cm^3^·cm/(cm^2^·s·Pa), respectively. This is consistent with Dong et al.’s analysis of PA6 hydrogen permeation behavior under different pressures [8], which indicated a decreasing trend in permeability coefficient with increasing experimental pressure.

For non-soluble gases like H_2_, the permeability coefficient decreases with increasing test pressure [51]. This phenomenon is attributed to the compression of the polymer and the compaction of voids as the test pressure rises [20,51]. Consequently, the reduction in free volume within the material more significantly hinders the diffusion of H_2_ molecules, decreasing the hydrogen permeability coefficient. Fumitoshi et al. [52] observed that under high-pressure hydrogen environments, the crystallinity of polymer materials is further enhanced, which impedes hydrogen diffusion. Therefore, as experimental pressure increases, the diffusion coefficient of the material decreases.

### 4.4. Analysis of H_2_ Diffusion Mechanism in Graphene/PA6 Composite Systems

Distribution of Free Volume:

The formula is as follows (5):

(5) $$FFV=\frac{V-V_{o}}{V}=\frac{V_{f}}{V}$$

In the equation, V represents the volume of the polymer unit cell, $V_{o}$ denotes the volume occupied by the polymer within the unit cell, and $V_{f}$ signifies the free volume within the polymer unit cell.

**Figure 11:**
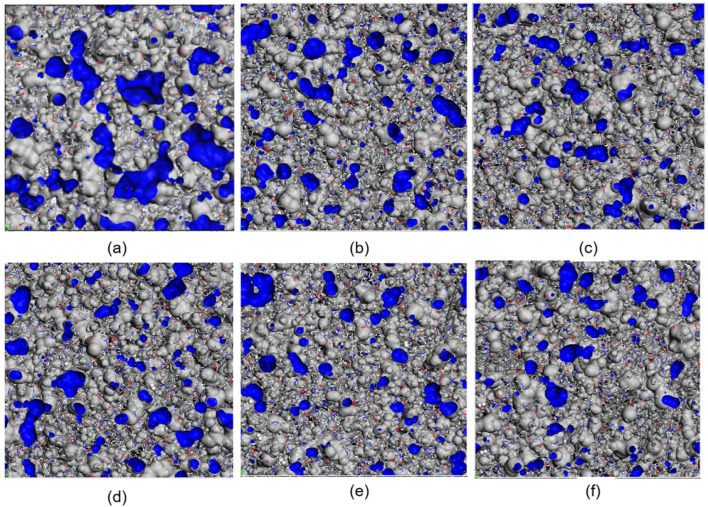
FFV of Different Systems at 298 K and 0.1 MPa: (**a**) PA6, (**b**) 3 wt%, (**c**) 4 wt%, (**d**) 5 wt%, (**e**) 6 wt%, and (**f**) 7 wt% (Blue area: free volume distribution of the composite).

According to Fox and Flory’s [53] free volume theory, the volume of a polymer can be divided into the occupied volume by polymer chains and the free volume not occupied by polymer chains. Hydrogen molecules (H_2_) can only diffuse within the free volume of the polymer. Changes in the free volume provide more space for H_2_ diffusion within the polymer, thereby affecting the diffusion coefficient. This study employed the hard probe method to investigate the free volume of graphene/PA6 composite systems at different proportions. The probe radius corresponds to the van der Waals radius of H_2_ (1.40 Å), representing the spatial extent of H_2_ diffusion within the polymer. The distribution of free volume for various graphene/PA6 composite systems at 298 K and 0.1 MPa is illustrated in Figure 11, where the blue regions indicate the distribution of free volume within the composite materials.

It can be observed that the blue area representing the FFV is the largest for pure PA6. Upon adding the 3 wt% graphene, the blue area decreases and then fluctuates with increasing graphene content. The FFV initially decreases and then increases, reaching a minimum when the graphene content is 4 wt%. However, when graphene content exceeds 5%, the blue area begins to increase again. These results indicate that the addition of graphene restricts the movement of polymer chains within PA6, thereby disrupting the continuity of “voids” within PA6 to some extent and reducing the polymer’s FFV. This limitation consequently restricts H_2_ diffusion within the polymer matrix, enhancing the gas barrier properties of the polymer. As graphene content continues to increase, new “voids” form between the filler and PA6, resulting in an increase in free volume. This provides additional channels for gas diffusion, deteriorating the barrier properties and consequently increasing the diffusion coefficient while reducing the barrier performance.

**Figure 12:**
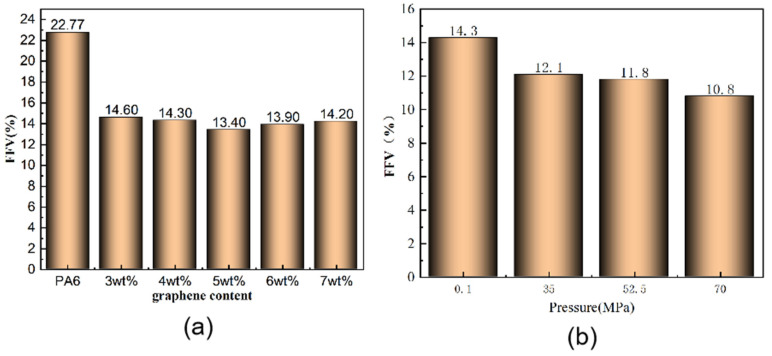
(**a**) FFV for different filler contents at 298 K and 0.1 MPa; (**b**) FFV of 4% Graphene/PA6 at four different pressure conditions.

Figure 12 shows the FFV calculated from simulation results to further analyze the impact of free volume fraction (FFV). Figure 12a depicts the FFV of different composite systems at 298 K and 0.1 MPa. The trend aligns with the distribution of the blue regions in Figure 11.

Figure 12b illustrates the FFV of 4% graphene/PA6 under four different pressure conditions. It is evident from the figure that the FFV decreases gradually with increasing pressure. This phenomenon arises because pressure reduces the distance between molecules in the composite material, thereby slightly diminishing the size and number of pores within the system.

**Figure 13:**
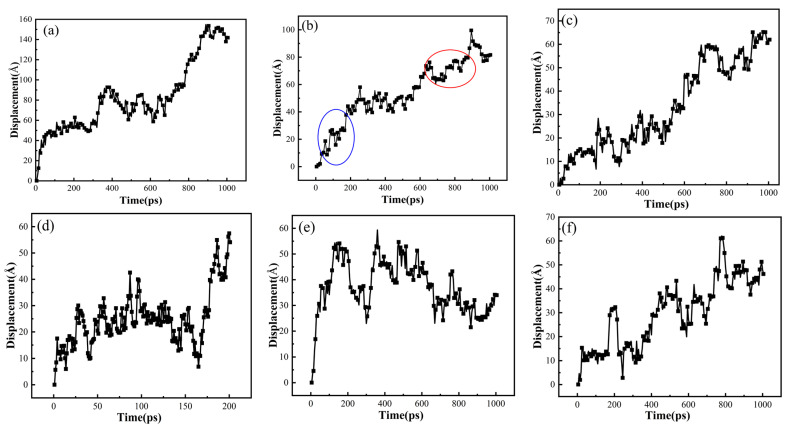
Trajectories of H_2_ in different systems at 298 K and 0.01 MPa: (**a**) PA6, (**b**) 3 wt%, (**c**) 4 wt%, (**d**) 5 wt%, (**e**) 6 wt%, and (**f**) 7 wt% (blue ellipse: hopping, red circle: vibration).

The trajectories of H_2_ in various graphene/PA6 systems were studied at 298 K and 0.1 MPa to investigate the motion of H_2_ molecules within polymers. Figure 13a illustrates that H_2_ diffusion in the PA6 system follows a “hop and jump” mechanism, where H_2_ molecules move between adjacent pores in a relatively short time. Over time, H_2_ molecules move from their initial positions, increasing overall displacement. Upon adding 3% graphene, Figure 13b shows that H_2_ molecules in the graphene/PA6 composite exhibit a distinct “hop and vibration” motion, indicating that the presence of graphene effectively hinders gas molecules. When hydrogen molecules encounter graphene during their motion, they cannot pass through directly, thus deviating from their original diffusion path; instead, they hop back and forth within the free volume. In Figure 13b, blue ellipse represent hopping, while red circle indicate vibration.

With the increase of graphene content, the vibration of H_2_ molecules within the polymer decreases, and there is a noticeable increase in hopping frequency, as shown in Figure 13d. Moreover, as the graphene content reaches 6 wt% and 7 wt%, the movement trajectory of H_2_ exhibits a pattern of “forward hopping” + “backward hopping”, with the backward hopping displacements being larger.

**Figure 14:**
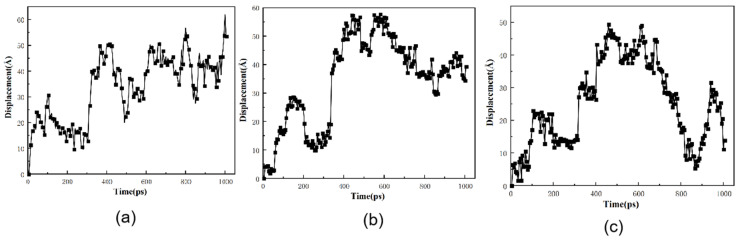
Displacement of H_2_ in 5% graphene/PA6 at different pressures: (**a**) 35 MPa, (**b**) 52.5 MPa and (**c**) 70 MPa.

The movement trajectories of H_2_ in the 4 wt% graphene/PA6 system under different pressures are shown in Figure 14. When the pressure reaches 35 MPa, a distinct pattern of forward “hopping” + backward “hopping” trajectories is observed, indicating that pressure enhances molecular motion while also affecting the distribution of free volume. As the pressure further increases to 70 MPa, the available space for hydrogen molecule movement decreases. Although pressure promotes molecular motion, the polymer chains become more distorted during movement. The more intense the local segment movements, the greater the probability of molecular transitions. Consequently, H_2_ molecules exhibit longer and more frequent hopping displacements.

## 5. Conclusions

Using molecular dynamics (MD) simulations, the dissolution, diffusion, and permeability characteristics of H_2_ in PA6 were studied across varying graphene contents (3 wt%–7 wt%), temperatures (233 K–358 K), and pressures ranging from 0.1 to 70 MPa. The following conclusions were drawn from this study.

(1) The addition of graphene restricts the movement of polymer chains and disrupts the continuity of “pores” within the polymer. When H_2_ moves within the polymer composite system, it influences the formation of free volume to a certain extent. Compared to pure PA6, under conditions of 0.1 MPa and 298 K, the permeability coefficient of the 5 wt% graphene/PA6 composite system decreases by 54.6%.

(2) Temperature has no significant effect on the solubility coefficient of graphene-added composite systems. The diffusion coefficients of materials in each system increase with rising temperature, and the increase in diffusion coefficient becomes more pronounced at higher temperatures. The permeability coefficients of each system decrease with increasing pressure, and within the same system, the permeability coefficient decreases as pressure increases.

(3) Additionally, the FFV and movement trajectories of H_2_ in each system were studied. FFV directly influences the diffusion coefficients of materials: the larger the FFV, the larger the diffusion coefficient. H_2_ diffusion in graphene/PA6 composite systems follows a “vibration + leap” mechanism. In the 3 wt% graphene/PA6 composite system, H_2_ exhibits noticeable vibration. In the 7 wt% graphene/PA6 composite system, H_2_ displays both significant “forward leaps” and “backward leaps”. With increasing pressure, H_2_ in the 4 wt% graphene/PA6 system shows longer leap distances and higher frequencies.

## Figures and Tables

**Table 1:** Dissolution, diffusion coefficients, and permeability coefficients of H_2_ in different material systems.

Material System	Solubility (cm^3^·cm^−3^·Pa^−1^)	Diffusion Coefficient (cm^2^/s)	Permeation Coefficient (cm^3^·cm/(cm^2^·s·Pa))
PA6	1.32 × 10^−6^	1.785 × 10^−6^	2.356 × 10^−12^
3%graphene/PA6	3.33 × 10^−7^	9.34 × 10^−7^	3.11 × 10^−13^
4%graphene/PA6	3.22 × 10^−7^	9.19 × 10^−7^	2.95 × 10^−13^
5%graphene/PA6	3.02 × 10^−7^	8.09 × 10^−7^	2.44 × 10^−13^
6%graphene/PA6	3.02 × 10^−7^	8.56 × 10^−7^	2.58 × 10^−13^
7%graphene/PA6	3.28 × 10^−7^	8.99 × 10^−7^	2.95 × 10^−13^

**Table 2:** Diffusion coefficients D (cm^2^/s) of six systems at different test temperatures under 70 MPa.

Material System	PA6	3 wt%	4 wt%	5 wt%	6 wt%	7 wt%
233 K	3.14 × 10^−7^	2.89 × 10^−7^	1.97 × 10^−7^	3.05 × 10^−7^	3.33 × 10^−7^	3.35 × 10^−7^
298 K	7.35 × 10^−7^	6.9 × 10^−7^	6.09 × 10^−7^	5.28 × 10^−7^	6.32 × 10^−7^	7.10 × 10^−7^
358 K	2.28 × 10^−6^	1.02 × 10^−6^	1.08 × 10^−6^	1.26 × 10−6	1.36 × 10^−6^	1.34 × 10^−6^

**Table 3:** Permeability Coefficients of 6 Systems at 298 K under Different Pressures (cm^3^·cm/(cm^2^·s·Pa)).

	PA6	3 wt%	4 wt%	5 wt%	6 wt%	7 wt%
0.1 MPa	2.35 × 10^−12^	3.11 × 10^−13^	2.74 × 10^−13^	2.44 × 10^−13^	2.58 × 10^−13^	2.95 × 10^−13^
35 MPa	1.44 × 10^−12^	2.54 × 10^−13^	2.15 × 10^−13^	2.34 × 10^−13^	2.41 × 10^−13^	2.88 × 10^−13^
52.5 MPa	1.39 × 10^−12^	2.56 × 10^−13^	2.00 × 10^−13^	2.05 × 10^−13^	2.15 × 10^−13^	2.42 × 10^−13^
70 MPa	9.70 × 10^−13^	2.29 × 10^−13^	1.96 × 10^−13^	2.04 × 10^−13^	1.90 × 10^−13^	2.33 × 10^−13^

## Data Availability

The data presented in this study are contained within the article and are also available upon request from the corresponding author.
